# The Corset as a Factor in Pelvic Diseases

**Published:** 1892-12

**Authors:** 


THE CORSET AS A FACTOR IN PELVIC DISEASES.—(*American Gynecological Journal*, August, 1892.) By G. R. West, M. D. This article deals at length with the injuries from what he calls the habitu.l use of the corset, exerting its influence on the osseous system first, the thorax second, the abdomen third and the pelvis fourth. If the author had confined himself to the abuse of the corset his paper would probably do more good. "In all probability the Indian and the Chinese and the uncorseted woman breathe with the abdominal movement of man." But it must be remembered that these various ladies are unaccustomed to the modern skirt, especially that used in England, which is heavy to begin with, and, with its trimming of braid and possibly the fashionable leather binding, is quite a burden to carry. Do away with the corset—the sensible corset, we mean—and nine times out of ten this skirt, with various other accessories, will hang fr.m the waist, and surely the pelvic contents will suffer far more than if the corset were used. We think that the corset is a natural accompaniment for the dress of modern civilization, and, until we return to the simplicity of the Greek or the Japanese, or the innocence of Eve or Pocahontas, we will have the corset always with us —*Int. Med. Mag.*

